# Prosthetic Hygiene

**Published:** 1890-06

**Authors:** J. Hall Lewis

**Affiliations:** Washington, D. C.


ARTICLE II.

### PROSTHETIC HYGIENE

BY PROF. J. HALL LEWIS, D.D.S., WASHINGTON, D. C.

[Read before the Washington City Dental Society, March 18th, 1890.

It seems about settled at the present day, among the medical profession, that this body of ours is, in health, but a conglomerate mass of minute active organisms, each living its own life and performing its allotted functions towards nourishing and sustaining the system as a whole; and that disease is but a riot or rebellion of one or more colonies of these organisms, who by their temporary refusal to perform their proper task, pervert, for the time being, the function of some part or organ sufficiently important to be felt by the entire system. Just as the strike of a large body of wage-earners, and their refusal to perform *their* appointed tasks, affects not only the factories in which they are employed, but also the community of which they are a part, and, indirectly, the nation to which they owe allegiance.

The microscope tells us that we live in the midst of myriads of micro-organisms—that the air we breathe, the water we drink, the food we consume, all contain, in immense numbers, microbic socialists and free-lances, ever ready to enter our systemic manufactory and there preach treason and sedition to the minute atoms which make up the bodily tissues, thus turning them from their allegiance to the central power of life and development, and by their inflammatory doctrines cause local strikes, organic dead-locks, and constitutional revolutions.

It is believed that at present only nine of the innumerable varieties of micro organisms around us have been conclusively proven to possess the property of directly causing disease; but it is expected that very soon the list of these will be materially enlarged, and physicians of all creeds are now combatting disorders from this stand-point. The homœopath riddles the deadly microbe with countless sugar-shot; the allopath crushes him with a ten-grain capsule; the hydropath drowns him; the faith-curist prays for him; and all unite in treating him as a common enemy.

Attention is not confined, however, to simply destroying the intruder, but also to preventing his ingress; and *preventive* medicine is daily receiving more and more attention from the scientist, who is already overwhelming us with warnings and suggestions regarding the barring-out of this omnipresent disease-producing marauder.

The air we breathe, he says, should be filtered through antiseptic gauze; the water we drink must be boiled, or else, as some prefer, liberally adulterated with the most potent "Dew of Old Kentucky." Before imbibing milk, it it should be punched with care. If we approach the lips of sweet sixteen with osculatory intent, a bug-killer must intervene; for upon the very pinnacle of Cupid's Brow may crouch a gigantic monster of full one ten-thousandth of an inch in length, ready to spring upon us, to our undoing. The old-fashioned feather duster is interdicted, and our office furniture should be cleansed by means of a cloth and a small boy, in order that the aforesaid cloth and boy may collect the micro-organisms, instead of ourselver.

To come down to solid facts, it is at present believed that bacteria abound; that some of these are producers, or carriers of disease and that they should be prevented *in every possible way* from gaining an entrance into our bodies Also, the production, or rather propagation and multiplycation of these microbes, is favored by a condition of immobility, of comparative warmth, and by the presence of a sustaining fluid. Again, that the disease-producers are dangerous, in proportion to their numbers, the system seem ing to tolerate the presence of a comparative few, but being unable to withstand the onslaught of a multitude of the same variety.

It is a fact, a sad fact, that the very great majority of artificial dentures worn at the present day are constructed of vulcanite, as the basal material. When this rubber is carefully and skillfuly vulcanized, the resulting product is porous in structure, containing numberless gas-cells, opening one into the other, thus forming multitudes of spaces, which may be likened to hollow beads strung upon and opening into a connecting tube. If the indurating process be ignorantly or hurriedly performed, then this ever-present condition of porosity may be very materially increased.

Again, as regards the *process* of vulcanization. It is sufficient for the scope of this paper to say, that it is a chemical reaction, brought about by the fact that the hydrogen of the caoutchouc, when under heat, has a greater affinity for the sulphur present than for the carbon, with which it was formerly united, and sulphurated hydrogen is thus formed, which *escapes* as a well-known malodorous gas.

It is thus seen, that the soft rubber, while heating, gives up sulphur and hydrogen, and gets *nothing* in return; it, therefore, contracts or lessens in bulk.

*But*, the artificial teeth, to which the rubber fastens itself through the medium of the pins, are, during the process of hardening, held immovably by the plaster investment; they cannot, therefore, follow the contracting rubber; hence, a *space* must exist between the porcelain and the vulcanite.

Again, if gum sections are used, there are five joints in a full denture, which are decidedly not fluid tight, and which are often protected posterially from discolonzation by cement, or plaster of paris, which afterwards dissolves, or washes out, leaving additional space between the porcelain and rubber.

The preparation for the mouth of such a set of artificial teeth, by the average dentist, consists in polishing the lingual surface of the rubber more or less thoroughly, and of cleansing the palatal aspect more or less *poorly*. Thus, it is seen, we have a denture, the basal material of which is naturally porous, and in which a space exists between the porcelain and the rubber, which space is often enlarged by a veritable trench, back of each joint.

Some dentists instruct their patients to remove their dentures, at night; others advise their retention at all times; still, others give no instructions whatever; and very many patients do as they please, without regard to instructions.

Thus, days, months, years, go by. Sooner or later, the polish given the lingual aspect of the denture is worn away; with it, the effect of surface condensation disappears and the softer and naturally porus structure of the rubber is exposed. The vulcanite material itself, as well as the spaces heretofore shown to exist under the porcelain, soon become thoroughly saturated with the fluids of the mouth, which are loaded with decomposing organic matter. Now, I ask, could a more perfect bacterial culture apparatus be conceived by human ingenuity

Here, we have a constant and equitable temperature of about 98°; perfect rest and quiet; an ever-present fluid medium; food of sufficient quantity and variety to tickle the palate of the most fastidious microbe epicure; and, lastly, the germs and spores in the food materials, and in the air which we at times breathe through the mouth. Hence, every space, every vulcanite pore becomes the seat of a thriving bacterial community—the bacilli hob-nob with the spiro-chetæ; the micrococci with their cock-eyed brothers, the spirillæ; and all increase and multiply, after their kind, until the denture is but a seething mass of corruption, and, according to the germ theory of the disease, is in a fit state to introduce, possibly, fatal disorders into the wearer's system, at the very moment when that system is, perhaps, least able to withstand the onslaught.

Not only to the wearer, but also to those in his immediate vicinity, is this a source of possible danger, for every word which the possessor of such a denture utters is charged with microcobic life, and, at the very moment that he kindly inquires after your good health, he may fire into your lungs a load of phthisical germs, as you take breath to return the compliment.

And now let us see what attention the advanced physician, or the antiseptic surgeon, gives to this subject. He examines the tongue, the fæces, the urine, of his fever-stricken, or diptheretic patient; he scans closely his food and drink; but, does he ever cast a single glance at, or give a moment's thought to, the artificial denture which stands at the side-entrance, so to speak, of the lungs, and which guards the very initial process of digestion?

Can it be doubted, that the chances of recovery of the invalid, suffering from lack of assimilative power, are lessened, when the food introduced into his stomach, and the remedies employed to combat his disease, are loaded with the contents of these multitudinous cess-pools? Or, what hope is there for the recovery of the patient, afflicted with some bowel disorder, if, with his nourishment, is poured into his digestive tract immense numbers of the very organisms positively said to produce his particular disease?

The physician, in short, pays no attention to the artificial teeth of his patients, leaving these substitutes to the care of the dentist, who is supposed to know all about them, and to give all needed advice as to their proper treatment.

So far, this subject has been considered solely from what might be called a microscopical stand-point; and it is undoubtedly true, that many members of our profession believe that the practical utility of microscopical investigation is overestimated, and that, in fact, about these little bugs there is a great deal of humbug. Let us, then, examine the question, from the view of these gentlemen; with the unaided senses of sight and smell.

A vulcanite denture is credited with causing, in many cases, excessive absorption of the underlying tissues, though in any given case is ever present the possibility that the same would have occurred under a metallic base. Congestion of the neighboring mucous membrane, at times violent inflammation and ulceration, a persisently heated sensation in the parts, chronic tonsilitis, and excessive flow of saliva—all these, and many more troubles, have, time and again, been traced directly to the presence of *old* vulcanite dentures. Also very often is the wearer an object of discomfort, or even disgust, to the associates, who have a *keen* sense of smell; for, be it known, that we possess this sense in different degrees, and are "near-smelled" quite as often as near-sighted.

A porous material, as bulky as the average full denture, saturated with decomposing matter, even provided the wearer use all ordinary care to cleanse it, cannot but possess a disgusting odor, and be an object of discomfort to the surrounding tissues, and, certainly, an exciting cause of at least *local* disease, in favoring systemic conditions.

Now, whether the germ theory of spreading disease be over-estimated or not, if the other evils mentioned be a possible result of wearing unclean dentures (as is most assuredly the fact), do you think that we, as members of a scientific and progressive profession, do our full duty to our patients, in view of the slight notice we take of the artificial substitutes, after they are once inserted in the mouth? I am afraid that, as a rule, we do not give that thought and attention to this subject that its importance demands.

It would be considered almost criminally careless for the dentist, after thoroughly filling all cavities of decay in a patient's mouth, to dismiss the patient with the teeth heavily coated with calculus; yet the same dentist, after carefully removing all foreign matter from the remaining natural organs, polishing and purifying these, until, with the surrounding tissues, they are perfectly sweet and healthy—that same dentist may often, with lofty disdain, take in his immaculate fingers a five-year old rubber plate, with its accompanying adornments of tartar, nicotine, decomposing food, and bacterial swarms, and snap it in the mouth, mentally thanking heaven that he is *not* a mechanical dentist, and has nothing to do with such horrid objects.

The very great majority of dentists, however, err in this direction, simply because of carelessness. They have never given the matter any amount of thought; allow the student, or office-boy, to vulcanize and prepare the case, and, after inserting it, with a few general directions, dismiss it entirely from their minds, when they dismiss the patient.

That very little study has been given to this subject is evidenced by the fact that, while countless numbers of papers have been written on "Root-filling," "Bridging"—in fact, every common operation of general dentistry and dental hygiene—I have failed, after a very diligent search, to find more than *one* writing on the subject under consideration, and that one simply sounded a note of warning, and made no attempt to combat the evils mentioned, or to prevent the propagation of the bacteria described as being present in an artificial denture.

It is to those dentists, then, who have given no thought to this phase of prosthetic dentistry that I offer the following suggestions, with the hope that thereby sufficient interest may be aroused to cause these ideas to be tried, improved upon, the results noted and given to the profession:

A "continuous gum" denture is by far the cleanest and purest of all forms of dental substitution; and the wearer can, by proper attention, keep such a denture absolutely free from decomposed food products and bacterial formations. It seems, however, that this material possesses certain peculiarities militating against its general use, besides the fact, that it is inadmissible in the great majority of partial dentures.

Next in desirability, from a hygienic stand-point, is the denture of gold, with the teeth fastened by means of vulcanite.

This method is applicable to partial as well as full dentures, is the strongest and least bulky of substitutes in general use, and seems more readily constructed by the dentist possessing average prosthetic skill than the continuous gum, and can be kept comparatively free from oral accumulations, though not to the extent possible in the case of the latter material. Pink rubber should connect the teeth to the gold plate; for, as it contracts less in vulcanizing than the other varieties, it, therefore, fits more snugly against the porcelain and gold.

If gum sections are used, the joints should be flat and solid, and without posterior protection, as pink rubber will not discolor them. Only the amount of rubber absolutely necessary should be present, and it should be covered by a wide rim of gold at the lingual aspect of the plate, extending up as far as, and as close as possible to, the pins of the teeth; thus reducing to a minimum the amount of material capable of absorbing the oral fluids and of harboring bacteria.

By all just and proper means should we endeavor to induce our patients to have their dentures constructed of either of the two materials described above; that is, of the so-called continuous gum, or of the gold and rubber combination. As, however, in spite of all talk against it, it is assuredly true that vulcanite is used in the very great majority of cases, it behooves us, as honorable servants of our patients, and as members of a scientific and progressive profession, to use this substance with due regard to their health and comfort, and in such a manner as to remove or counteract, as far as lies within, us, its possible baneful effects, whether local or systemic.

As before stated, while the material, in its very nature, is porous, great care must be used, in vulcanizing, that the heat be not carried to "too high" a point, else, from over-rapid elimination of sulphurated hydrogen, increased porosity will unavoidably result. To this end, the vulcanizing apparatus should be closely watched by some one possessing more knowledge and discrimination than the office-boy; the thermometer tested from time to time, and the mercury-bath examined; for, at times, the packing above this bath disintegrates, the metal, from its volatility, escapes, and the thermometer, though itself perfect, may thus register the degree of heat incorrectly.

The heat should not be continued *longer* than is requisite to properly indurate the rubber; for the *unnecessary* loss of hydrogen and sulphur results in greater contraction, and thus enlarges the unavoidable space between the porcelain and vulcanite. Pink rubber should be used in the more bulky portions of the dentures, and as generously as permissible, with a proper regard to strength, in order that the above-mentioned space may be reduced to a minimum.

*All* surfaces (the palatal, as well as the lingual, or buccal) of the vulcanite should receive as high a polish as the material will admit of, as this effects a surface condensation; thus more effectually preventing external deposits, and closing the pores to the entrance of the oral fluids and bacterial germs. But, inasmuch as this polish wears off, in the course of time, thus exposing the softer structure, it is "absolutely essential" that at certain stated intervals (say of six months) the denture should be returned to the dentist, to be thoroughly cleansed and re-polished by him. This, besides being a generally unthought-of source of revenue to the dentist, soon becomes a most desirable and highly appreciated process to the patient, because of the added comfort and cleanliness, the truth of which only needs a trial to demonstrate. The prosthetic dentist who fails to insist upon a semi-annual cleansing and polishing of the artificial teeth, is just as culpably careless of his patient's welfare as is the operative dentist who neglects to remove calculus and other deposits from the natural teeth of those under his charge.

But it has again and again been stated that the vulcanite is even at best an exceedingly porous material, and is, therefore, capable of receiving and retaining decomposing fluids "within its own substance," and, in spite of the most faithful "surface" cleansing given it by the wearer, or the dentists. In order to meet this hygienic difficulty, brought about by the structural peculiarities of the material under consideration, the artificial teeth should be nightly placed in an antiseptic or disinfectant fluid, there to remain until morning; which fluid should permeate the vulcanite structure, insinuate itself into the space between the base and porcelain, and into all other interstices present; there to prevent or retard the decomposing process, and render harmless the ptomanic alkaloids of the bacterial formations. As this fluid is to be used freely and continuously by the patient, it must, of necessity, be free from poisonous qualities, easily prepared, and of slight cost.

Disinfectants, or germicides, act by destroying bacterial life, and are not to be considered in this connection, because of their poisonous attributes, and other readily observed reasons.

We must use, then, one or more of the many antiseptics. whose value depend upon the fact that they "retard" bacterial growth, and, consequently, lessen the production of ptomanic matter, which is dangerous in proportion to its quantity.

As a result of considerable experimentation and research, I have come to the conclusion that the very best fluid, all things considered, for this purpose, is the solution made from "Seiler's Antiseptic Tablets," consisting of sodium, the silico-fluoride, bicarbonate-borate and chloride, also eucalyptol, thymol, and oil of gaultheria. The small cost of this allows of its being used freely by plate-wearers, and it serves the purposes intended most excellently.

In use, a bottle is filled with clear water, and the tablets dropped in, at the rate of one tablet to four tablespoonfuls of water. The artificial denture is cleansed, placed in a suitable vessel, and covered with the antiseptic solution, which may first be further adulterated with an equal bulk of water. A gallon of this fluid will cost your patient less than fifty cents, is easily made, is perfectly harmless, and imparts to the denture a sweetness and freshness which is simply delightful, and which can be obtained by no amount of ordinary surface-cleansing.

If to the ordinary solution a few drops of oil of gaultheria or cassia, or both, with a little zinc. catechu, be added, you have a fluid very pleasing to the eye and the palate, with very slight additional cost.

In conclusion, gentlemen, should the patient once become accustomed to using this, or a similar antiseptic fluid, you may rest assured that he will not depart from the habit thus formed, in sickness or in health; the mucous membrane of the mouth will be given an opportunity to take "its" rest at night; the stomach will not receive a load of disease-producing germs at every mouthful of food, the case of "rubber sore mouth" in your practice will become exceedingly rare; and the breath of the wearer, instead of reeking with the stench-producing results of putrefaction and decomposition, will be laden with the balmy odors of cinnamon and of checkerberry; and the wearer and his friends will rise up and call you blessed; for you have made him, orally at all events, cleanly—and cleanliness, you know, is a kin to godliness.—"Archives of Dentistry."

